# Origin of the Yeast Whole-Genome Duplication

**DOI:** 10.1371/journal.pbio.1002221

**Published:** 2015-08-07

**Authors:** Kenneth H. Wolfe

**Affiliations:** UCD Conway Institute, School of Medicine & Medical Science, University College Dublin, Dublin, Ireland

## Abstract

Whole-genome duplications (WGDs) are rare evolutionary events with profound consequences. They double an organism’s genetic content, immediately creating a reproductive barrier between it and its ancestors and providing raw material for the divergence of gene functions between paralogs. Almost all eukaryotic genome sequences bear evidence of ancient WGDs, but the causes of these events and the timing of intermediate steps have been difficult to discern. One of the best-characterized WGDs occurred in the lineage leading to the baker’s yeast *Saccharomyces cerevisiae*. Marcet-Houben and Gabaldón now show that, rather than simply doubling the DNA of a single ancestor, the yeast WGD likely involved mating between two different ancestral species followed by a doubling of the genome to restore fertility.

## Abstract 2

The ancient whole-genome duplication in the baker's yeast lineage was described almost 20 years ago; in this Primer, one of its discoverers assesses the implications of evidence that it was preceded by hybridization between two species.

The unicellular baker’s yeast *Saccharomyces cerevisiae* was the first eukaryote to have its genome sequenced, using the first generation of automated sequencing machines and before the advent of the whole-genome shotgun approach. The sequencing was done during the period between 1990 and 1996 by an international consortium that included many small European laboratories, one of which was mine. Each laboratory was given a “tranche” of about 30 kb to sequence, and when you had completed that chunk, you could apply for another one. We were paid €2 per base pair. Progress meetings, chaired energetically by André Goffeau [1], were held every six months to ensure that the project remained on track. At these meetings, each group would make a 5-minute presentation about the genes they had found in their current chunk. The presentations were often tedious, enlivened only by the occasional exigency for André to reassign pieces of DNA from the sequencing tortoises to the hares. But as the project progressed, a pattern began to emerge: many of the chunks were similar to other chunks. The first clone that I sequenced happened to contain the centromere of chromosome II, and I noticed that a gene beside it had a paralog beside the centromere of chromosome IV [2]. My second chunk, from chromosome XV, contained four genes that had four paralogs, in the same order, on chromosome I [3].

When the complete genome was released in April 1996, we were able to identify 55 large duplicated blocks of this type, ranging in size from three to 18 duplicated genes (Fig 1) [4]. Two observations indicated that the duplications were quite old: the average amino acid sequence identity between the gene pairs was only 63%, and within each block only about 25% of the genes were actually duplicated, the others being single copy. This pattern suggested that the whole block was initially duplicated, and then many individual genes were deleted. Two other observations suggested that the blocks were remnants of duplicated chromosomes that had become rearranged during evolution: there were almost no overlaps between the blocks, and the orientation of each pair of blocks was conserved relative to the centromeres and telomeres. This layout of blocks was consistent with duplication of the whole genome followed by both extensive deletion of single genes and genome rearrangement solely by the process of reciprocal translocation between chromosomes [4]. Under this hypothesis, there had been an ancient whole-genome duplication (WGD), and the 55 blocks that we could identify were simply the most duplicate-dense regions that still survived without evolutionary rearrangement.

**Fig 1:**
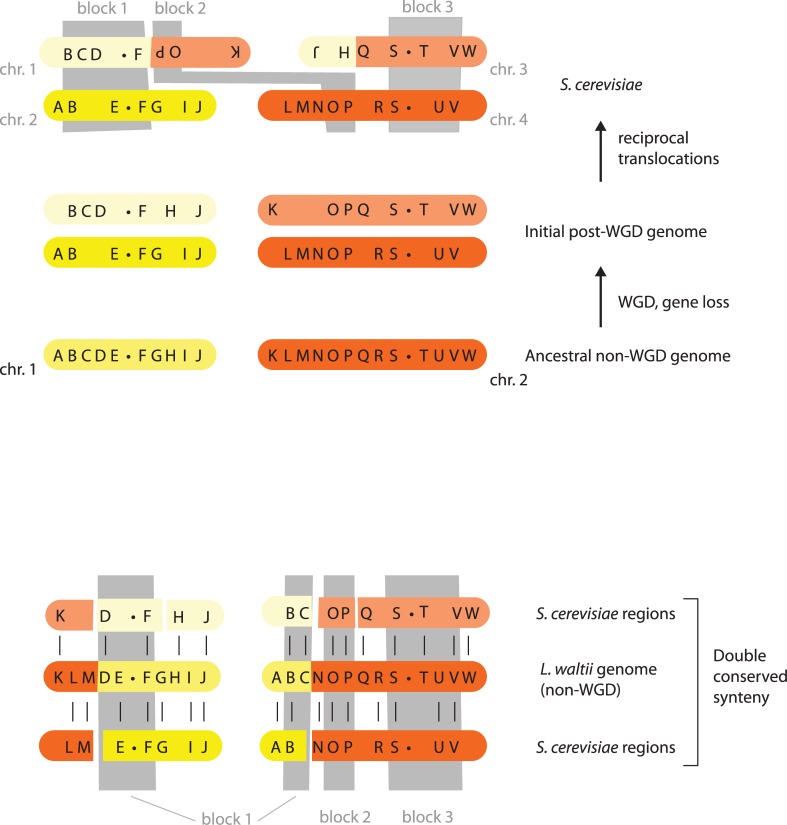
A simple model of WGD, gene loss, and synteny relationships. The upper panel shows how duplicated blocks were initially identified using only genes that remain in duplicate in *S*. *cerevisiae* [4]. The lower panel shows how additional data from non-WGD yeasts such as *Lachancea waltii* [5] allowed the parts of the genome that were not initially allocated to blocks to be placed into pairs, providing a duplication map that covered the whole *S*. *cerevisiae* genome. Letters A–W represent genes, and dots represent centromeres. Only two chromosomes (yellow and brown) are shown.

The hypothesis of a WGD in *S*. *cerevisiae* was confirmed in 2004 when three groups sequenced the genomes of species that had branched off from this lineage before the WGD occurred [5–7]. These non-WGD genomes had a “double conserved synteny” relationship with the *S*. *cerevisiae* genome—that is, instead of each pair of duplicated regions, they had a single region containing all the genes in a merged order (Fig 1). This discovery allowed the entire genome of *S*. *cerevisiae* to be mapped into pairs of regions via their double conserved synteny with the non-WGD species, even if the pairs retain no duplicated genes, thus filling the gaps between the initial map of 55 duplicated blocks. These analyses proved that the WGD encompassed the entire genome of *S*. *cerevisiae* and showed that its 16 centromeres fall into eight ancestral pairs that are syntenic with centromeres of the non-WGD species. It therefore appeared that the WGD turned an eight-chromosome ancestor into a 16-chromosome descendant. From this complete map, we now know that among the 5,774 protein-coding genes of *S*. *cerevisiae*, there are 551 pairs of duplicated genes (ohnologs) that were formed by the WGD and that about 144 chromosomal rearrangements scrambled the genome after the WGD [8,9]. We also know that the WGD is not confined to *Saccharomyces* but occurred in the common ancestor of six genera, some of which diverged from each at an early stage when more than 4,000 genes were still duplicated, leading to later losses of different gene copies in different lineages [10].

What were the molecular events that caused the WGD? It is relatively easy to draw a diagram summarizing the history of each chromosomal region (Fig 2), but it is much more difficult to specify the provenance of the intermediate molecules and the timescales involved. Two alternative scenarios can describe the steps in Fig 2. In both scenarios, event 1 is a DNA replication, and cells W and Z are each capable of mating (they are respectively a non-WGD haploid and a post-WGD haploid). The key question is whether the DNA molecules labeled X and Y existed in (1) two different cells of the same species or (2) two cells of two different species. Scenario 1 is called autopolyploidization, in which case event 1 corresponds to a simple cell division and event 2 is a mating between gametes from the same species or some other form of cell fusion. Scenario 2 is called allopolyploidization or hybridization, in which case event 1 is a speciation and event 2 is an interspecies mating or cell fusion. If event 2 was a mating, then an additional step such as deletion of one allele at the *MAT* locus is necessary to convert cell Z from a nonmating zygote to a mating gamete—but it is not essential that this additional step occurred immediately after event 2. In fact, a long delay in which cell Z replicated mitotically for many generations could be useful because it could allow reproductive isolation from cells of type W to build up. Eventually (event 3), mating between two post-WGD haploid cells of type Z can produce a post-WGD diploid like cell ZZ, which is the state in which *S*. *cerevisiae* is normally found in nature.

**Fig 2:**
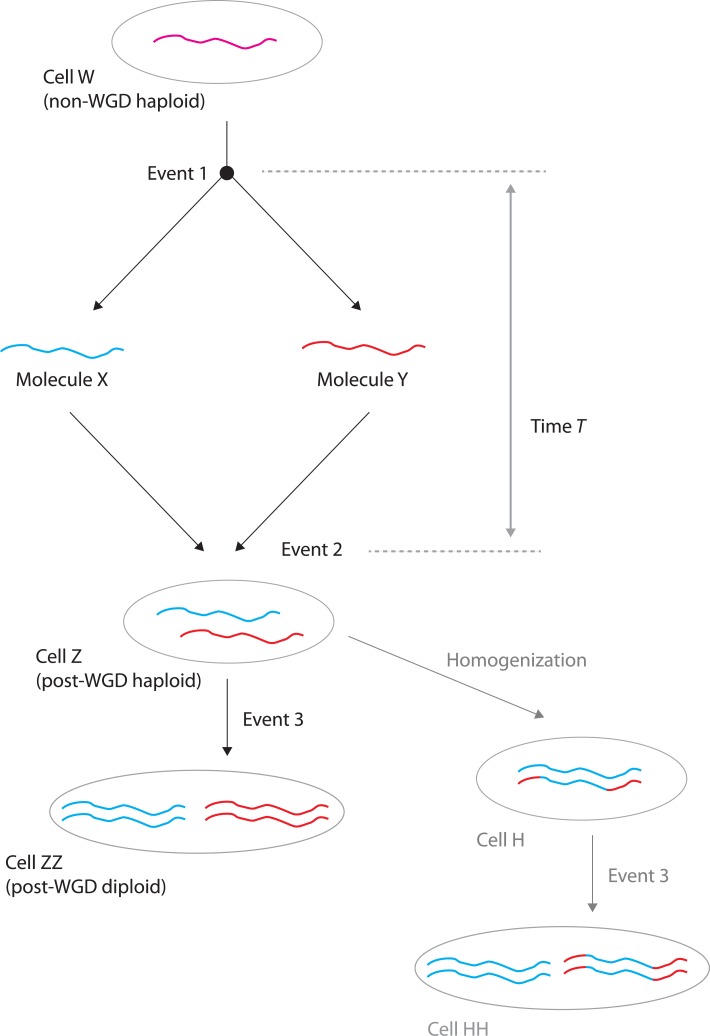
Tracing the history of a single chromosomal region. See text for details. In an allopolyploidization, the red and blue chromosomes are called homeologs.

The major difference between these two scenarios is the amount of time (*T*) that elapsed between events 1 and 2: was it a few generations or millions of years? In scenario 1, molecules X and Y must be identical, whereas in scenario 2 they could have any level of sequence divergence from minimal to extensive, and they could also differ by chromosomal rearrangements. It has been difficult to design tests that could differentiate between these scenarios, but an analysis of the inferred order of genes along molecules X and Y did not find any rearrangements and so did not rule out scenario 1 [9]. However, it has been frustrating that we could not pin down the details of this crucial phase of yeast evolution, which gave birth to many pairs of genes with substantially divergent functions [11–15].

In this issue of *PLOS Biology*, Marcet-Houben and Gabaldón now report strong evidence in support of interspecies hybridization (scenario 2) as the source of the two subgenomes in post-WGD species [16]. By phylogenetic analysis using state-of-the-art methods, they show that molecules X and Y have phylogenetic affinities to two different non-WGD lineages that they call the KLE and ZT clades. The KLE clade (*Kluyveromyces*, *Lachancea*, and *Eremothecium*) is the group of non-WGD species that was sequenced in 2004 [5–7]. The ZT clade (*Zygosaccharomyces* and *Torulaspora*) is a separate, more recently studied non-WGD lineage [17,18]. Previous phylogenetic studies using supertrees or concatenated data suggested that the ZT clade is sister to the post-WGD clade, with the KLE clade being an out-group to them both [17,19,20]. The new analysis [16] made trees for each gene individually and found that, although the majority of genes in post-WGD species do cluster phylogenetically with the ZT clade as expected, a significant minority (about 30%) instead either cluster with the KLE clade or form an outgroup to a KLE + ZT clade. This phylogenetic heterogeneity was not noticed before because the KLE signal is only present in a minority of genes, and it is swamped by the ZT signal in methods that try to place the post-WGD clade at a single point on the tree.

Marcet-Houben and Gabaldón interpret this phylogenetic heterogeneity as evidence that the two post-WGD subgenomes have separate origins, one from the ZT clade and the other from an unidentified lineage that is an outgroup to KLE + ZT. Under the simplest hypothesis of hybridization, we might then expect that phylogenetic trees constructed from ohnolog pairs should show one *S*. *cerevisiae* gene grouping with the ZT clade and the other grouping with the KLE clade, but in fact, most ohnolog pairs group with each other, with the ZT clade as their closest relative [16]. The authors’ explanation for these two results—an excess of ZT-like ohnolog pairs, and an excess of ZT-like genes in the whole genome (which is mostly singletons)—is that the post-WGD genomes have been affected by biased gene conversion that preferentially replaced some KLE-derived sequences with copies of the homeologous ZT-derived sequences, homogenizing these regions and obliterating their signal of KLE ancestry.

The hybridization proposed by Marcet-Houben and Gabaldón makes a lot of sense in terms of what we know about the biology of yeast interspecies hybrids. Many yeast strains, most notably those used in commercial settings where stress tolerance is important, have turned out to be interspecies hybrids. For instance, the yeast used to brew lager (*S*. *pastorianus*) is a hybrid between *S*. *cerevisiae* and *S*. *eubayanus* [21,22], and many other combinations of genomes from different species of *Saccharomyces* have been found in nature [23]. These interspecies hybrids are usually infertile (unable to sporulate) because the two copies (homeologs) of each chromosome that they contain are too dissimilar to pair properly during meiosis [24–26]. One simple way to restore fertility is to double the genome, allowing each chromosome to pair with an identical partner instead of trying to pair with the homeolog. In this model, cell Z changes from being a nonmater (effectively diploid) to a mater (effectively haploid—perhaps by deletion of a *MAT* allele), then two cells of type Z mate to produce cell ZZ (diploid), and cell ZZ is able to go through meiosis and make spores with twice the DNA content of cell W. Thus, one hypothesis that Marcet-Houben and Gabaldón propose is that event 2 was an interspecies mating and event 3 was a restoration of fertility by genome doubling, with a possible interval of many mitotic generations between these two events. Alternatively, they hypothesize that event 2 may have been an interspecies fusion of diploid cells, obviating the need for a separate event 3.

The obscuring of the phylogenetic signal of hybridization by subsequent gene conversions [16] is consistent with the known genome structures of some interspecies hybrids. The yeasts *Pichia sorbitophila* [27] and *Candida orthopsilosis* [28] are both interspecies hybrids, but in each case extensive homogenization of parts of the genome has occurred. This process of homogenization has been called overwriting, loss of heterozygosity, or gene conversion by different groups. It leaves the number of chromosomes unchanged (equal to the sum of the numbers of chromosomes in the two incoming subgenomes) but involves the replacement of sequences in one subgenome by sequences copied from the other subgenome (cell H in Fig 2). Homogenization could occur on scales as small as a few hundred base pairs (gene conversion) or as large as whole chromosome arms (break-induced replication). In the latter case, even differences in gene order such as inversions between the parental species could be ironed out.

The discovery that the yeast WGD was an allopolyploidization adds complexity to what initially seemed to be a simple story of duplication. If an interspecies hybrid such as *P*. *sorbitophila* with a partly homogenized genome developed two mating types and these could mate to form a diploid that could sporulate efficiently, the result would be a species with a genome resembling the inferred progenitor of the post-WGD clade (cell HH in Fig 2). Allopolyploidy answers some old questions about why genes were retained in duplicate if their sequences were identical (answer: they weren’t identical), what the immediate selective advantage of the post-WGD cell was (answer: hybrid vigor), and how the post-WGD lineage became reproductively isolated from the pre-WGD lineage (answer: delay between events 2 and 3). But it also raises new questions about homogenization (how much of the genome? how often? why is it biased?) and about the mechanism of restoration of fertility (why is event 3 so rare, apparently happening only once in the budding yeast family even though event 2 happened quite often?).

Ancient WGDs have been detected right across the eukaryotic tree of life, including in animals, ciliates, fungi, and, most prominently, plants [29–32]. If extensive gene conversion can obscure the traces of allopolyploidization in yeast genomes, one might wonder how many of these other ancient WGDs also began as interspecies hybridizations. In fact, there is evidence from plants that gene conversion acts continually to homogenize ohnolog pairs [32,33] and that hybrid plants can show preferential retention of DNA from one parent over the other [34] similar to the situation in *P*. *sorbitophila* [27]. Detecting the yeast hybridization in the presence of these obscuring factors required both good luck and good timing: good luck that a reference species closer to one parent than to the other had been sequenced and good timing that the hybrid was sampled before all traces of its hybrid origin had faded away. These fortunate circumstances may not hold for ancient hybridizations in other eukaryotes, but as a famous golfer once said, “The harder I practice, the luckier I get.” Detecting that they are hybridizations may become possible with exhaustive sampling of possible parental lineages and the use of sensitive phylogenomic methods of the type introduced by the authors [16].
